# Mortality in the United States — Provisional Data, 2023

**DOI:** 10.15585/mmwr.mm7331a1

**Published:** 2024-08-08

**Authors:** Farida B. Ahmad, Jodi A. Cisewski, Robert N. Anderson

**Affiliations:** 1 National Center for Health Statistics, CDC.

**Summary**

**What is already known about this topic?**

Provisional death estimates provide an early indication of shifts in mortality trends and can guide public health policies and interventions intended to reduce mortality.

**What is added by this report?**

More than 3 million persons died in the United States in 2023. The overall age-adjusted death rate in 2023 was 6.1% lower than in 2022. The overall death rate was highest among non-Hispanic Black or African American persons. The number of deaths from COVID-19 was 68.9% lower than in 2022.

**What are the implications for public health practice?**

Timely and actionable data can guide public health policies and interventions for populations experiencing higher mortality.

## Abstract

Final annual mortality data from the National Vital Statistics System for a given year are typically released 11 months after the end of the calendar year. Provisional data, which are based on preliminary death certificate data, provide an early estimate of deaths before the release of final data. In 2023, a provisional total of 3,090,582 deaths occurred in the United States. The age-adjusted death rate per 100,000 population was 884.2 among males and 632.8 among females; the overall rate, 750.4, was 6.1% lower than in 2022 (798.8). The overall rate decreased for all age groups. Overall age-adjusted death rates in 2023 were lowest among non-Hispanic multiracial (352.1) and highest among non-Hispanic Black or African American persons (924.3). The leading causes of death were heart disease, cancer, and unintentional injury. The number of deaths from COVID-19 (76,446) was 68.9% lower than in 2022 (245,614). Provisional death estimates provide an early signal about shifts in mortality trends. Timely and actionable data can guide public health policies and interventions for populations experiencing higher mortality.

## Introduction

The National Center for Health Statistics’ (NCHS) National Vital Statistics System (NVSS) collects and reports annual mortality statistics using U.S. death certificate data. Because of the time needed to investigate certain causes of death and to process and review death data, final annual mortality data for a given year are typically released 11 months after the end of the calendar year. Provisional data, which are based on preliminary death certificate data sent to NCHS, provide an early estimate of deaths before the release of final data. NVSS routinely releases provisional mortality data for all causes of death, including deaths involving COVID-19.* This report presents an overview of provisional U.S. mortality data for 2023, including a comparison with death rates from 2022 (*1*). Provisional death estimates provide an early indication of shifts in mortality trends and can guide public health policies and interventions intended to reduce mortality among populations experiencing higher mortality.

## Methods

### Data Source

This report analyzed provisional NVSS death certificate data for deaths occurring among U.S. residents in the United States during January–December 2023.^†^ NCHS tabulated the number and rates of overall deaths and COVID-19 deaths by age, sex, and race and ethnicity (categorized as non-Hispanic Asian [Asian], non-Hispanic American Indian or Alaska Native [AI/AN], non-Hispanic Black or African American [Black], non-Hispanic Native Hawaiian or Pacific Islander [NH/PI], non-Hispanic White [White], Hispanic or Latino [Hispanic], non-Hispanic persons of more than one race [multiracial], and unknown). NCHS coded the causes of death according to the *International Classification of Diseases, Tenth Revision,* which details disease classification and the designation of underlying cause of death^§^ (*2*). COVID-19 death counts and rates include deaths for which COVID-19 is listed on the death certificate as an underlying or contributing cause of death.^¶^ Leading causes of death were ranked by counts based on underlying cause of death (*3*). Data in this report exclude deaths among residents of U.S. territories and foreign countries. Age was unknown for 71 (<0.01%) decedents, and race and ethnicity were unknown for 10,068 (0.33%).

### Data Analyses

To describe the trend in deaths during a given year, the number of deaths were calculated for each week from all causes and from COVID-19. Age-adjusted rates were calculated for deaths overall and by sex, and race and ethnicity. Crude death rates were calculated by age. The population data used to calculate death rates are July 1, 2023, estimates based on the blended base produced by the U.S. Census Bureau (*4*,*5*). Unless otherwise specified, comparisons made in the text among rates are statistically significant (p<0.05 using a z-test). R software (version 4.0.3; R Foundation) was used to conduct all analyses. This activity was reviewed by CDC and was conducted consistent with applicable federal law and CDC policy.**

## Results

### Overall Measures

**FIGURE 1:**
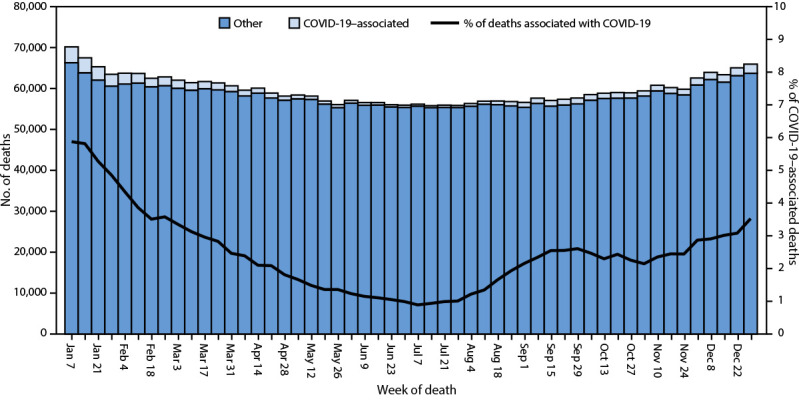
Provisional* number of COVID-19–associated deaths^†^ and other deaths and percentage of deaths associated with COVID-19, by week of death — National Vital Statistics System, United States, 2023 * National Vital Statistics System provisional data for 2023 are incomplete. Data from December 2023 are less complete because of reporting lags. These data exclude deaths that occurred in the United States among residents of U.S. territories and foreign countries. ^†^ Deaths with confirmed or presumed COVID-19 as an underlying or contributing cause of death, with *International Classification of Diseases, Tenth Revision* code U07.1.

In 2023, a total of 3,090,582 deaths occurred in the United States (Table). The age-adjusted rate was 750.4 deaths per 100,000 population, a decrease of 6.1% from 798.8 in 2022. The number of deaths was highest during the week ending January 7 (68,965) and during the week ending December 30 (65,257) (Figure 1). In 2023, death rates per 100,000 were lowest among persons aged 5–14 years (14.7) and highest among persons aged ≥85 years (14,285.8), similar to patterns in 2022 (Table). Death rates decreased from 2022 to 2023 for all age groups (although not significantly for ages 0–4 years). Age-adjusted death rates in 2023 were higher among males (884.2) than among females (632.8), and lower than in 2022 (males = 954.5; females = 666.1).

**TABLE:** Provisional* number and rate of total deaths and COVID-19–associated deaths, by demographic characteristics — National Vital Statistics System, United States, 2022–2023

Characteristic	No. of deaths (rate^†^)				p-value of rate difference	
	2022		2023		Total^§^	COVID-19–associated
	Total	COVID-19–associated^§^	Total	COVID-19–associated^§^		
**Total**	**3,279,857 (798.8)**	**245,614 (58.7)**	**3,090,582 (750.4)**	**76,446 (18.2**)	**<0.05**	**<0.05**
**Age group, yrs**						
<1	**20,553 (558.0)**	248 (6.7)	**20,140 (552.0)**	99 (2.7)	**0.27**	<0.05
1–4	**4,156 (28.0)**	162 (1.1)	**4,058 (27.3)**	68 (0.5)	**0.21**	<0.05
5–14	**6,239 (15.3)**	210 (0.5)	**6,005 (14.7)**	71 (0.2)	**<0.05**	<0.05
15–24	**35,232 (79.5)**	649 (1.5)	**33,708 (76.8)**	142 (0.3)	**<0.05**	<0.05
25–34	**74,369 (163.4)**	2,406 (5.3)	**67,427 (148.1)**	428 (0.9)	**<0.05**	<0.05
35–44	**111,605 (255.4)**	5,220 (11.9)	**105,288 (237.2)**	899 (2.0)	**<0.05**	<0.05
45–54	**183,284 (453.3)**	12,242 (30.3)	**166,707 (411.7)**	1,896 (4.7)	**<0.05**	<0.05
55–64	**417,541 (992.1)**	30,627 (72.8)	**376,433 (899.4)**	5,725 (13.7)	**<0.05**	<0.05
65–74	**668,581 (1,978.7)**	53,361 (157.9)	**627,589 (1,809.4)**	13,628 (39.3)	**<0.05**	<0.05
75–84	**824,903 (4,708.2)**	67,235 (383.7)	**798,153 (4,345.3)**	23,253 (126.6)	**<0.05**	<0.05
≥85	**933,291 (14,389.6)**	73,250 (1,129.4)	**885,003 (14,285.8)**	30,236 (488.1)	**<0.05**	<0.05
Unknown	**103 (—)**	4 (—)	**71 (—)**	1 (—)	**NA**	NA
**Sex**						
Female	**1,560,607 (666.1)**	112,559 (47.4)	**1,473,817 (632.8)**	37,127 (15.4)	**<0.05**	<0.05
Male	**1,719,250 (954.5)**	133,055 (73.7)	**1,616,765 (884.2)**	39,319 (22.1)	**<0.05**	<0.05
**Race and ethnicity**						
AI/AN, NH	**23,613 (947.9)**	2,127 (83.5)	**21,273 (830.6)**	480 (18.7)	**<0.05**	<0.05
Asian, NH	**89,591 (417.5)**	6,879 (32.0)	**85,763 (387.9)**	2,335 (10.7)	**<0.05**	<0.05
Black or African American, NH	**411,934 (1,002.8)**	28,854 (71.0)	**385,323 (924.3)**	6,823 (17.0)	**<0.05**	<0.05
NH/PI, NH	**4,592 (782.0)**	379 (64.3)	**4,461 (730.1)**	82 (13.8)	**<0.05**	<0.05
White, NH	**2,448,093 (822.2)**	180,533 (58.6)	**2,308,103 (778.1)**	60,860 (19.6)	**<0.05**	<0.05
Hispanic or Latino	**275,684 (614.7)**	25,167 (58.2)	**258,766 (559.0)**	5,393 (13.0)	**<0.05**	<0.05
Multiracial, NH	**16,904 (366.8)**	1,062 (25.1)	**16,825 (352.1)**	289 (7.0)	**<0.05**	<0.05
Unknown	**9,446 (—)**	613 (—)	**10,068 (—)**	184 (—)	**NA**	NA

**Abbreviations:** AI/AN = American Indian or Alaska Native; NA = not applicable; NH = non-Hispanic; NH/PI = Native Hawaiian or Pacific Islander.

* National Vital Statistics System provisional data for 2023 are incomplete. Data from December 2023 are less complete because of reporting lags. Data for 2022 are final. These data exclude deaths that occurred in the United States among residents of U.S. territories and foreign countries.

^†^ Deaths per 100,000 standard population. Age-adjusted death rates are provided overall and by sex and race and ethnicity.

^§^ Deaths with confirmed or presumed COVID-19 as an underlying or contributing cause of death, with *International Classification of Diseases, Tenth Revision* code U07.1.

**FIGURE 2:**
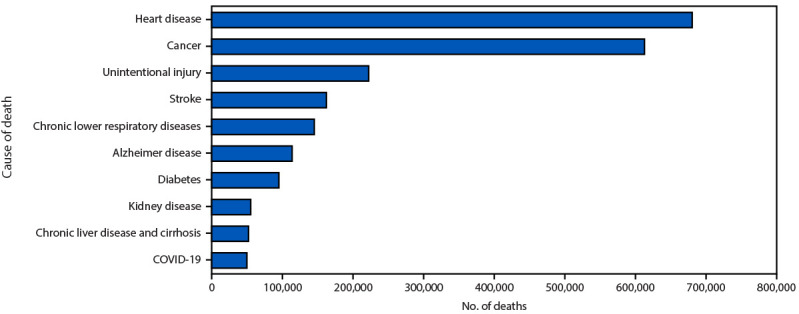
Leading underlying causes of death* — National Vital Statistics System, United States, 2023 * National Vital Statistics System provisional data for 2023 are incomplete. Data from December 2023 are less complete because of reporting lags. These data exclude deaths that occurred in the United States among residents of U.S. territories and foreign countries.

Age-adjusted death rates per 100,000 in 2023 differed by race and ethnicity and decreased from 2022 to 2023 for all groups (Table). Rates were lowest among multiracial (352.1) and highest among Black persons (924.3). The three leading causes of death were heart disease (680,909 deaths), cancer (613,331), and unintentional injury (222,518) (Figure 2).^††^ COVID-19, listed as the underlying cause in 49,928 deaths during 2023, ranked as the 10th leading underlying cause of death.

### COVID-19 Measures

During 2023, COVID-19 was listed as the underlying or contributing cause of 76,446 deaths (18.2 per 100,000), a 68.9% decrease from 245,614 (58.7 per 100,000) in 2022 (Table). The COVID-19 death rate decreased from 2022 to 2023 for all age groups. As with deaths overall, the age-adjusted COVID-19–associated death rate per 100,000 among males (22.1) was higher than that among females (15.4). COVID-19–associated death rates decreased from 2022 to 2023 for all racial and ethnic groups (Table).

## Discussion

The estimated age-adjusted death rate, 750.4 per 100,000 persons, was 6.1% lower in 2023 than in 2022 (798.8) (*1*). Death rates were highest among males, older adults, and Black persons. The highest weekly numbers of overall deaths and COVID-19 deaths occurred during early January and late December. The leading causes of death in 2023 were heart disease, cancer, and unintentional injury. COVID-19, the fourth leading cause of death in 2022 became the 10th leading cause in 2023. COVID-19 was the underlying cause for 1.6% of all deaths in 2023, decreasing from 5.7% (186,552 deaths) in 2022. Deaths from heart disease decreased in 2023 compared with 2022 (702,880 deaths), and deaths from cancer increased from 2022 (608,371).

Overall death rates and COVID-19–associated death rates decreased from 2022 to 2023 for all demographic groups (but not significantly for ages 0–4 years). Although the overall and COVID-19 death rates decreased from 2022 to 2023 for persons aged ≥85 years, rates for this group remained higher than those for all other age groups. Overall and COVID-19–associated death rates decreased for all racial and ethnic groups. In 2023, White and AI/AN persons had the highest COVID-19–associated death rate (19.6 and 18.7, respectively), a shift from 2020−2022 when COVID-19 death rates were highest among AI/AN persons (*1*,*6*,*7*).

### Limitations

The findings in this report are subject to at least three limitations. First, data are provisional, and numbers and rates might change as additional information is received. For example, previously published provisional counts of deaths for 2022 were slightly lower than the final counts of deaths for 2022 (*1*,*8*). This finding is due to certain causes of deaths (e.g., unintentional injury deaths) that are known to be reported with a more substantial lag so that the final death count will likely be higher than reported currently (*9*). Described differences in rates and mortality trends might be underestimates. Second, timeliness of death certificate submission can vary by jurisdiction. As a result, the national distribution of deaths might be affected by the distribution of deaths reported from jurisdictions reporting later, which might differ from those in the United States overall. For example, late reporting from a jurisdiction with a large number of deaths in a particular demographic group could substantially increase the number and rate of deaths for the United States. Finally, potential for misclassification of certain categories of race (e.g., AI/AN or Asian) and Hispanic ethnicity reported on death certificates (*10*) exists; thus, death rates for some groups might be underestimated or overestimated.

### Implications for Public Health Practice

This report provides an overview of provisional mortality in the United States during 2023. Provisional death estimates can give researchers and policymakers an early signal about shifts in mortality trends and provide actionable information sooner than do the final mortality data. These data can guide public health policies and interventions that are intended to reduce mortality.

## Footnotes

* NVSS provisional mortality data are available at https://wonder.cdc.gov. NVSS COVID-19 surveillance data are available at https://www.cdc.gov/nchs/nvss/deaths.htm.

† Based on death records received and processed as of July 21, 2024, for deaths occurring in the United States among U.S. residents. Data included in this analysis reflect >99% of deaths that occurred in 2023.

§
https://www.cdc.gov/nchs/nvss/manuals/2a-sectioni-2021.htm

¶ The underlying cause of death is the disease or injury that initiated the train of morbid events leading directly to death. A contributing cause of death is a disease or injury also listed on the death certificate, that is not classified as the underlying cause of death.

** 45 C.F.R. part 46, 21 C.F.R. part 56; 42 U.S.C. Sect. 241(d); 5 U.S.C. Sect. 552a; 44 U.S.C. Sect. 3501 et seq.

†† The number of deaths attributed to unintentional injury were tabulated using the internal NVSS mortality database and might not match publicly available provisional counts for deaths for unintentional injury because provisional data on injury-related causes of death are publicly released with a lag of 6 months from the date of death. This delay accounts for the additional time typically needed to report injury-related death certificates, and the final 2023 death count might be higher than noted in this report.
